# The efficacy of preoperative MRI features in the diagnosis of meningioma WHO grade and brain invasion

**DOI:** 10.3389/fonc.2022.1100350

**Published:** 2023-01-18

**Authors:** Jun Jiang, Juan Yu, Xiajing Liu, Kan Deng, Kaichao Zhuang, Fan Lin, Liangping Luo

**Affiliations:** ^1^ Department of Radiology, Health Science Center, Shenzhen Second People’s Hospital, The First Affiliated Hospital of Shenzhen University, Shenzhen, China; ^2^ Philips Healthcare, China International Center, Guangzhou, China; ^3^ Medical Imaging Center, The First Affiliated Hospital of Jinan University, Guangzhou, China

**Keywords:** brain invasion, WHO grade, meningioma, MRI, diagnosis

## Abstract

**Objective:**

The preoperative MRI scans of meningiomas were analyzed based on the 2021 World Health Organization (WHO) Central Nervous System (CNS) Guidelines, and the efficacy of MRI features in diagnosing WHO grades and brain invasion was analyzed.

**Materials and methods:**

The data of 675 patients with meningioma who underwent MRI in our hospital from 2006 to 2022, including 108 with brain invasion, were retrospectively analyzed. Referring to the WHO Guidelines for the Classification of Central Nervous System Tumors (Fifth Edition 2021), 17 features were analyzed, with age, sex and meningioma MRI features as risk factors for evaluating WHO grade and brain invasion. The risk factors were identified through multivariable logistic regression analysis, and their receiver operating characteristic (ROC) curves for predicting WHO grades and brain invasion were generated, and the area under the curve (AUC), sensitivity and specificity were calculated.

**Results:**

Univariate analysis showed that sex, tumor size, lobulated sign, peritumoral edema, vascular flow void, bone invasion, tumor-brain interface, finger-like protrusion and mushroom sign were significant for diagnosing meningioma WHO grades, while these features and ADC value were significant for predicting brain invasion (P < 0.05). Multivariable logistic regression analysis showed that the lobulated sign, tumor-brain interface, finger-like protrusion, mushroom sign and bone invasion were independent risk factors for diagnosing meningioma WHO grades, while the above features, tumor size and ADC value were independent risk factors for diagnosing brain invasion (P < 0.05). The tumor-brain interface had the highest efficacy in evaluating WHO grade and brain invasion, with AUCs of 0.779 and 0.860, respectively. Combined, the variables had AUCs of 0.834 and 0.935 for determining WHO grade and brain invasion, respectively.

**Conclusion:**

Preoperative MRI has excellent performance in diagnosing meningioma WHO grade and brain invasion, while the tumor-brain interface serves as a key factor. The preoperative MRI characteristics of meningioma can help predict WHO grade and brain invasion, thus facilitating complete lesion resection and improving patient prognosis.

## Introduction

Meningioma is one of the most common brain tumors, representing 37.6% of primary intracranial tumors (1). Although the 2021 WHO Guidelines for the Classification of Tumors (5th Edition) propose a greater reliance on genetic testing for grading, morphological classification remains divided into 15 pathological subtypes and grades 1, 2 and 3 (2). The tumors are diverse in biological characteristics in terms of different pathological subtypes and grades, and the tumor recurrence rate is closely related to the WHO grade and completeness of surgical resection. WHO grade 1 meningiomas have a very low recurrence rate after total resection, but there is a higher tendency for recurrence as the WHO meningioma grade increases. The five-year recurrence rates after total resection for meningiomas have been reported to be 7%~23% for WHO grade 1, 50%~55% for WHO grade 2, and 72%~78% for WHO grade 3; subtotally resected tumors usually have poor prognosis (3). The 2016 4th edition of the WHO CNS guidelines included brain invasion as a diagnostic criterion for WHO grade 2 meningiomas and modified WHO grade 1 meningiomas with brain invasion to atypical meningiomas, increasing the incidence rate of WHO grade 2 meningiomas by 1% to 10% (4). With regard to the analysis of data collected from 2016 and 2022, WHO grade 2 meningiomas accounted for 1/5 to 1/3 of all meningiomas (5). Notably, in comparison with meningiomas without brain invasion, meningiomas with brain invasion exhibit aggressive behaviors, an increased recurrence rate (6), three times more bleeding intraoperatively, and an increased risk of postoperative seizures as well as postoperative bleeding (7).

However, there is a gross underestimation of meningioma brain invasion (7). Due to incomplete surgical resection and incomplete sampling, the incidence of brain invasion is underestimated, and the postoperative recurrence rate is high (8). Because of a lack of attention and emphasis on preoperative imaging assessments, the specific imaging signs of brain invasion are still unclear (9), with very few previous imaging studies including brain invasion as an independent factor (10–15).

Therefore, based on the 2021 edition of the Central Nervous System (CNS) WHO guidelines, this paper discusses the diagnostic value of clinical and MRI-specific features for WHO grade and brain invasion in meningioma to provide adequate information for preoperative preparations, improve the resection efficacy for the tumor and invaded brain tissue, and reduce recurrence and mortality rates while improving patient prognosis.

## Materials and methods

### Subjects

The research proposal has been reviewed and approved by the ethics committee of our hospital, with the approval number 20190910. All data from meningiomas resected in our hospital from 2006 to 2022 were analyzed retrospectively. The inclusion criteria were as follows: (1) preoperative MRI examination performed; and (2) meningioma confirmed through routine pathology and immunohistochemistry of surgically resected lesion tissue. The exclusion criteria were as follows: (1) pathological findings that diagnosed brain invasion in meningioma but did not describe the specimen as containing brain tissue; (2) preoperative treatment; and (3) previous surgical resection for the same tumor. A total of 675 meningioma cases were included in the study.

### Magnetic resonance imaging

A 3.0T (Siemens MAGNETOM Prisma) or 1.5T (Siemens MAGNETOM Avanto) MRI scanner was used, with 20- or 8-channel head coils. The sequences and scanning parameters were as follows: T1-weighted imaging (T1WI) (3T: repetition time (TR) 500 ms, echo time (TE) 7.4 ms, field of view (FOV) 320 mm×240 mm, slice thickness (ST) 6 mm; 1.5 T: TR 388 ms, TE 13 ms, FOV 199 mm×220 mm, ST 6 mm); T2-weighted imaging (T2WI) (3T: TR 5000 ms, TE 117 ms, FOV 220 mm×220 mm, ST 6 mm; 1.5 T: TR 4000 ms, TE 95 ms, FOV 220 mm×220 mm, ST 6 mm); fluid-attenuated inversion recovery (FLAIR) (3T: TR 9000 ms, TE 81 ms, FOV 220 mm×220 mm, ST 6 mm; 1.5 T: TR 8650 ms, TE 96 ms, FOV 220 mm×220 mm, ST 6 mm); diffusion-weighted imaging (DWI) (3T: TR 2900 ms, TE 98 ms, FOV 230 mm×230 mm, ST 6 mm; 1.5 T: TR 2900 ms, TE 97 ms, FOV 220 mm×220 mm, ST 6 mm); and T1-weighted postcontrast (T1C) (contrast agent: gadolinium borate; 3T: TR 600 ms, TE 7.6 ms, FOV 320 mm×240 mm, ST 1.5 mm; 1.5 T: TR 350 ms, TE 9.6 ms, FOV 199 mm×220 mm, ST 1.5 mm).

### Radiological data

**Figure 1:**
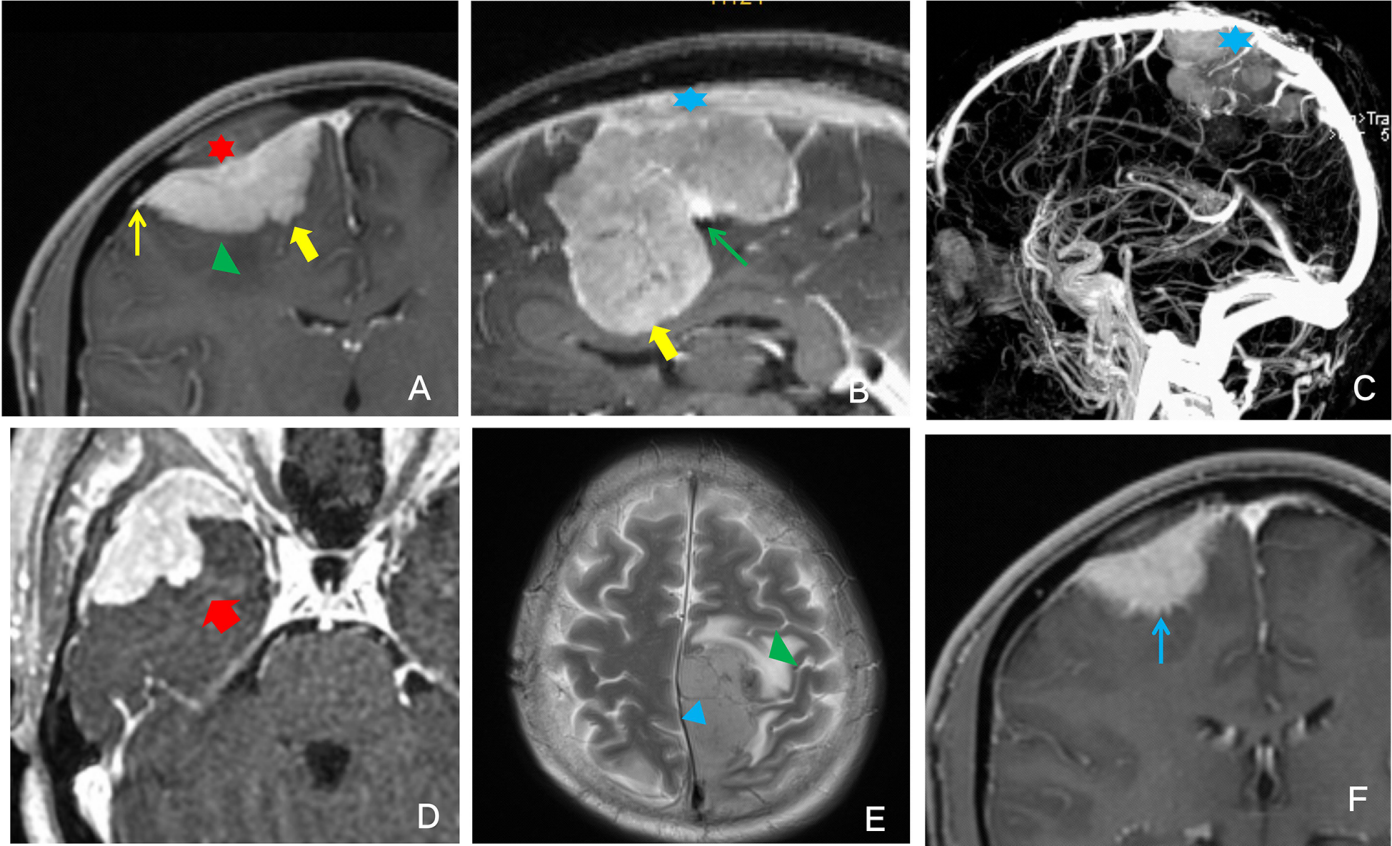
Illustrative example of the description of the analyzed imaging features. **(A)** Red star: bone invasion; green triangle: peritumoral edema; thin yellow arrow: dural tail sign; thick yellow arrow: finger-like protrusion. **(B)** Blue star: venous sinus invasion; thin green arrow: lobulated sign; thick yellow arrow: finger-like protrusion. **(C)** Blue star: venous sinus invasion. **(D)** The thick red arrow points to the enhanced signal as the mushroom sign. **(E)** Green triangle: peritumoral edema; blue triangle: vascular flow void. **(F)** Thin blue arrow: unclear tumor-brain surface.

An associate senior neuroimaging specialist (11 years of experience) and a senior specialist (16 years of experience) evaluated the images on the PACS workstation without knowing the pathological results, and any differences were resolved through discussion. The MRI scans were evaluated for meningioma features (T1WI signal intensity (SI), T2WI SI, degree and homogeneity of T1C, tumor size, lobulated sign, peritumoral edema, ADC value, vascular flow-void sign, dural tail sign, venous sinus invasion, bone invasion, tumor-brain interface, finger-like protrusions, and mushroom sign) (**Figure 1**).

The MRI signal was scored according to the Elster criteria (16): T1WI SI: 1 point: the signal is significantly lower than that of the cerebral gray matter and is close to that of the cerebrospinal fluid; 2 points: the signal is slightly below the cerebral gray matter signal; 3 points: the signal is close to the gray matter signal; 4 points: the signal is lightly higher than the gray matter signal; and 5 points: the signal is significantly higher than the gray matter signal and close to the fat signal. T2WI SI: 1 point: the signal is significantly lower than that of the gray matter and close to that of the bone cortex; 2 points: the signal is slightly lower than the gray matter signal; 3 points: the signal is close to the gray matter signal; 4 points: the signal is slightly higher than the gray matter signal; and 5 points: the signal is significantly higher than the gray matter signal and close to the cerebrospinal fluid signal. T1C enhancement degree: 1 point: significantly enhanced, enhanced SI close to that of fat; 2 points: moderately enhanced, enhanced SI slightly lower than that of fat; 3 points: mildly enhanced, enhanced SI lower than that of fat but higher than the gray matter signal.

The tumor size(volume) was measured by the software on PACS. The lobulated sign refers to an uneven, curved depression or convex change in the tumor surface margin. The vascular flow void sign referred to tumor vessels in which MRI could not collect blood flow signal, showing a low signal in the shape of a cord on T1WI and T2WI sequences. The dural tail sign manifested as tumor-adjacent meningeal enhancement, thickening, and distal thinning. Venous sinus invasion was evaluated on T2WI and T1C and observed as tumor adhesion to the venous sinus, invasion of the venous sinus, or complete occlusion of the venous sinus. Bone invasion could be clearly seen on T1C as an enhanced signal at the site of invasion. The tumor-brain interface refers to the tumor boundary. When tumor progression did not reach a certain degree, the tumor was separated from the brain tissue by the cerebrospinal fluid-vascular gap and arachnoid interface, and a low signal ring was present on T1C. When the low signal ring disappeared, the tumor-brain interface was considered unclear. Finger-like protrusions could be clearly shown on T1C, and the tumor border could be clearly observed with tumor tissue protruding in a finger-like pattern into the adjacent brain parenchyma. The mushroom sign was observed on T1C as an enhancing band of spherical tumor invading peripherally along the dural attachment; this sign is more distant, thicker and longer than the commonly seen dural tail sign, and the proximal cerebral surface is often more irregular and uneven.

### Histopathological data

All pathological findings were reinterpreted by two neuropathologists referring to the 2021 CNS WHO guidelines, and the morphological diagnosis was made using the “933” grading model (2, 4, 5), i.e., 9 WHO grade 1, 3 WHO grade 2 and 3 WHO grade 3, to determine the pathological grade of meningiomas and to diagnose cases of brain invasion (**Table 1**). Based on the latest 2021 5th guidelines, brain invasion otherwise benign meningiomas (BIOB) were classified as WHO grade 2 in this study. The diagnostic criteria for brain invasion were as follows (15) (1): HE-stained slides of the tumor-brain interface revealed irregular, tongue-like invasion into the brain parenchyma without soft meningeal involvement; (2) glial cell proliferation and neuronal degradation in the invaded brain tissue; and (3) positive immunohistochemical staining for GFAP in paraffin sections; brain invasion from the tumor was considered if any of the above criteria were met. In this study, the pathology report description of each case was required to include brain tissue; otherwise, the case was excluded from the study.

**Table 1:** Histopathological classification of meningioma [N(%)].

Histopathological classification	N(%)	Brain invasion	
		yes	no
**WHO grade 1/BIOB**	**610(90.4)**	**67(11.0)**	**543(89.0)**
Meningothelial meningioma	147(24.1)	23(34.3)	124(22.8)
Fibrous meningioma	127(20.8)	9(13.4)	118(21.7)
Transitional meningioma	249(41.8)	26(38.8)	223(41.0)
Psammomatous meningioma	41(6.7)	4(5.9)	37(6.8)
Angiomatous meningioma	29(4.8)	2(2.9)	27(4.9)
Microcystic meningioma	11(1.8)	2(1.4)	9(1.6)
Secretory meningioma	4(0.6)	1(1.4)	3(0.5)
Metaplastic meningioma	1(0.1)	0(0.0)	1(0.1)
Lymphoplasmacyte-rich meningioma	1(0.1)	0(0.0)	1(0.1)
**WHO grade 2**	**56(8.3)**	**32(57.2)**	**24(42.8)**
Atypical meningioma	49(87.5)	30(93.1)	19(79.1)
Chordoid meningioma	4(7.1)	1(3.1)	3(12.5)
Clear cell meningioma	3(5.3)	1(3.1)	2(8.3)
**WHO grade 3**	**9(1.3)**	**9(100.0)**	**0(0.0)**
Anaplastic meningioma	7(77.8)	7(77.8)	0(0.0)
Rhabdoid meningioma	1(11.1)	1(11.1)	0(0.0)
Papillary meningioma	1(11.1)	1(11.1)	0(0.0)
**Total**	**675**	**108(16.0)**	**567(84.0)**

WHO, World health organization; BIOB, Brain invasion otherwise benign meningiomas.

### Statistical analysis

SPSS software (v.26.0, IBM, USA) was used for statistical analysis, and Medcalc software (v.20.0.22, Solvusoft, USA) was used to generate the receiver operating characteristic (ROC) curves. Descriptive statistics were applied for age, sex, and meningioma MRI features using the results of the evaluation performed by the senior specialist, with continuous variables expressed as the mean ± standard deviation and categorical variables expressed as frequency distributions. All the characteristic parameters were analyzed in univariate logistic regression as factors for meningioma WHO grade and brain invasion, and the meaningful parameters were selected for multivariate logistic regression analysis. ROC curves of the selected parameters for the diagnosis of WHO grade and brain invasion were generated, and AUC, sensitivity and specificity were calculated. P value < 0.05 was considered statistically significant.

## Results

Among the 675 meningiomas cases, 543 (80.4%) were WHO grade 1, 123 (18.2%) were WHO grade 2, and 9 (1.3%) were WHO grade 3; 567 (84.0%) cases were meningiomas without brain invasion, and 108 (16.0%) were meningiomas with brain invasion. A consistency test was carried out on the data evaluated by the two neuroradiology experts, and the correlation coefficient ranged from 0.848 to 0.997, indicating good consistency between the two experts.

### Association of WHO grades with findings on radiological imaging

The clinical data and MRI features were compared between WHO grade 1 and WHO grade 2/3 lesions. Univariate logistic regression showed that age (P=0.258), T1WI (P=0.615), T2WI (P=0.617), degree of T1C (P=0.754), T1C enhancement homogeneity (P=0.869), ADC value (P=0.780), dural tail sign (P=0.384), and venous sinus invasion (P=0.062) were not associated with WHO grade. However, the male/female ratio was 56/76 for WHO grade 2/3 and 154/409 for WHO grade 1 (P=0.002). The mean size of WHO grade 2/3 tumors was larger than that of WHO grade 1 tumors (P=0.000). In addition, the lobulated sign, peritumoral edema, vascular flow void, bone invasion, unclear tumor-brain interface, finger-like protrusion, and mushroom sign were more common in WHO grade 2/3 tumors than in WHO grade 1 tumors (P<0.05; **Table 2**).

**Table 2:** Univariate analysis of features associated with WHO grades [N(%)].

Features		WHO 1	WHO 2/3	Exp(B)	P-value
N		543	132		
Age (years)		51.1 ± 12.7	49.6 ± 15.0	0.992	0.258
Sex	Male	154 (28.3)	56 (42.4)	0.537	0.002
	Female	389 (71.6)	76 (57.6)		
T1WI	1	11 (2.0)	4 (3.0)	0.911	0.615
	2	110 (20.2)	30 (22.7)		
	3	413 (76.0)	93 (70.4)		
	4	8 (1.4)	5 (3.7)		
	5	1 (0.1)	0 (0.0)		
T2WI	1	11 (2.0)	3 (2.2)	1.139	0.617
	2	40 (7.3)	10 (7.5)		
	3	302 (55.6)	68 (51.5)		
	4	169 (31.1)	45 (34.0)		
	5	21 (3.8)	6 (4.5)		
T1C	1	397 (73.1)	96 (72.7)	1.058	0.754
	2	128 (23.5)	30 (22.7)		
	3	18 (3.4)	6 (4.5)		
Enhancement homogeneity	heterog-eneous	362 (66.7)	87 (66.0)	0.967	0.869
	homog-eneous	181 (33.3)	45 (34.0)		
Tumor size (cm^3^)		26.0 ± 2.1	47.1 ± 2.6	1.822	0.000
Lobulated sign	yes	230 (42.4)	71 (53.8)	1.584	0.018
	no	313 (57.6)	61 (46.2)		
Peritumoral edema	yes	196 (36.1)	84 (63.7)	3.098	0.000
	no	347 (63.9)	48 (36.3)		
ADC value (/×10^-5^ mm^2^·s^-1^)		908.4 ± 165.8	877.7 ± 208.7	1.000	0.780
Vascular flow void	yes	95 (17.5)	39 (29.6)	1.978	0.002
	no	448 (82.5)	93 (70.4)		
Dural tail sign	yes	470 (86.5)	118 (89.3)	1.309	0.384
	no	73 (13.5)	14 (10.7)		
Venous sinus invasion	yes	191 (35.2)	58 (43.9)	1.444	0.062
	no	352 (64.8)	74 (56.1)		
Bone invasion	yes	63 (11.6)	50 (37.8)	4.646	0.000
	no	480 (88.4)	82 (62.2)		
Tumor-brain surface	clear	443 (81.5)	34 (25.8)	12.769	0.000
	unclear	100 (18.5)	98 (74.2)		
Finger-like protrusion	yes	10 (1.9)	38 (28.7)	21.547	0.000
	no	533 (98.1)	94 (71.3)		
Mushroom sign	yes	2 (0.4)	25 (18.9)	63.201	0.000
	no	541 (99.6)	107 (81.1)		

WHO, World Health Organization; T1WI, T1-weighted imaging; T2WI, T2-weighted imaging; T1C, T1-weighted postcontrast.

Multivariate logistic regression analysis revealed that the lobulated sign (OR 0.528; 95% CI 0.307~0.909; P=0.021), tumor-brain interface (OR 7.946; 95% CI 4.427~14.262; P=0.000), finger-like protrusion (OR 4.845; 95% CI 2.076~11.310; P=0.000), mushroom sign (OR 9.346; 95% CI 2.014~43.376; P=0.004), and bone invasion (OR 2.311; 95% CI 1.315~4.061; P=0.004) were independent risk factors for the diagnosis of meningioma WHO grade.

**Figure 2:**
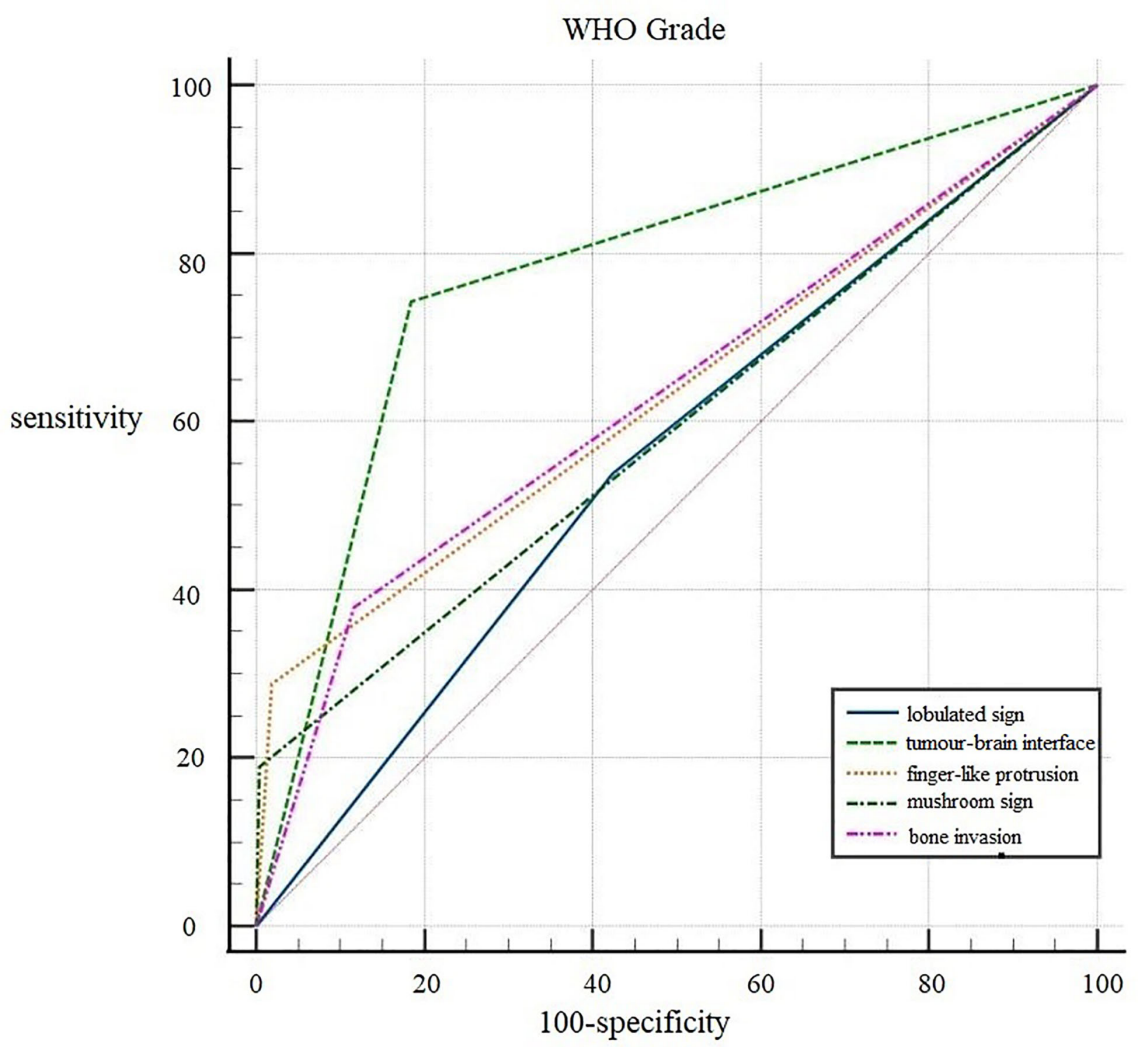
ROC curves of independent risk factors for WHO grade.

ROC curves were generated for the five independent risk factors for WHO grade: lobulated sign, tumor-brain interface, finger-like protrusion, mushroom sign, and bone invasion (**Figure 2**). The results showed that the tumor-brain interface had the highest diagnostic accuracy for WHO grade (AUC 0.779; 95% CI 0.746~0.810; sensitivity 0.742; specificity 0.816). Finally, a ROC curve was created for the fitted variable for WHO grade obtained by multivariate logistic regression (AUC 0.834; 95% CI 0.832-0.885; sensitivity 0.727; specificity 0.858), reflecting a strong diagnostic efficacy.

### Association of brain invasion with findings on radiological imaging

**Table 3** summarizes the associations among of age, sex, 15 imaging features and brain invasion. Univariate logistic regression analysis showed that age (P=0.331), T1WI (P=0.656), T2WI (P=0.933), degree of T1C (P=0.687), enhancement homogeneity (P=0.682), dural tail sign (P=0.773), and venous sinus invasion (P=0.077) were not associated with brain invasion. However, the male/female ratio was higher among meningiomas with brain invasion (48/60) than among meningiomas without brain invasion (162/405) (P=0.001). The meningiomas with brain invasion had a significantly larger mean size than the noninvasive meningiomas (P=0.000). The mean ADC values were lower for meningiomas with brain invasion than for meningiomas without brain invasion (P=0.008). In addition, the lobulated sign, peritumoral edema, vascular flow void, bone invasion, unclear tumor-brain interface, finger-like protrusion, and mushroom sign were more common in meningiomas with brain invasion than in meningiomas without brain invasion (P < 0.05; **Table 3**).

**Table 3:** Univariate analysis of features associated with brain invasion [N(%)].

Features		Brain invasion	Non-brain-invasion	Exp(B)	P-value
N		108 (16.0)	567 (84.0)		
Age (years)		49.9 ± 14.4	51.2 ± 13.0	0.992	0.331
Sex	male	48 (44.4)	162 (28.6)	0.500	0.001
	female	60 (55.6)	405 (71.4)		
T1WI	1	4 (3.7)	11 (1.9)	0.915	0.656
	2	22 (20.4)	118 (20.8)		
	3	79 (73.2)	427 (75.3)		
	4	4 (2.7)	10 (1.7)		
	5	0 (0.0)	1 (0.1)		
T2WI	1	3 (2.7)	11 (1.9)	1.012	0.933
	2	7 (6.4)	43 (7.5)		
	3	59 (54.6)	311 (54.8)		
	4	34 (31.4)	180 (31.7)		
	5	5 (4.6)	22 (3.8)		
T1C	1	78 (72.2)	415 (73.1)	1.081	0.687
	2	25 (23.1)	133 (23.4)		
	3	5 (4.6)	19 (3.3)		
Enhancement homogeneity	heterog-eneous	70 (64.8)	379 (66.9)	0.914	0.682
	homog-eneous	38 (35.1)	188 (33.1)		
Tumor size (cm^3^)		54.3 ± 2.5	25.6 ± 2.1	1.980	0.000
Lobulated sign	yes	62 (57.4)	239 (42.1)	1.850	0.004
	no	46 (42.6)	328 (57.8)		
Peritumoral edema	yes	73 (67.5)	207 (36.6)	3.627	0.000
	no	35 (32.4)	360 (63.4)		
ADC value (/×10^-5^ mm^2^ -s^-1^)		862.9 ± 165.8	910.2 ± 176.3	0.998	0.001
Vascular flow void	yes	35 (32.5)	99 (17.4)	2.267	0.000
	no	73 (67.5)	468 (82.6)		
Dural tail sign	yes	95 (87.9)	493 (86.9)	1.097	0.773
	no	13 (12.1)	74 (13.1)		
Venous sinus invasion	yes	48 (44.4)	201 (35.4)	1.457	0.077
	no	60 (55.6)	366 (64.6)		
Bone invasion	yes	49 (45.3)	64 (11.2)	6.527	0.000
	no	59 (54.6)	503 (88.8)		
Tumor-brain surface	clear	11 (10.1)	466 (82.2)	40.686	0.000
	unclear	97 (89.9)	101 (17.8)		
Finger-like protrusion	yes	38 (35.1)	10 (1.7)	30.237	0.000
	no	70 (64.9)	557 (98.3)		
Mushroom sign	yes	25 (23.1)	2 (0.3)	80.090	0.000
	no	83 (76.9)	565 (99.7)		

T1WI, T1-weighted imaging; T2WI, T2-weighted imaging; T1C, T1-weighted postcontrast.

Multivariate logistic regression analysis revealed that tumor size (OR 1.270; 95% CI 1.020~1.582; P=0.033), ADC value (OR 0.998; 95% CI 0.996~1.000; P=0.043), lobulated sign (OR 0.309; 95% CI 0.150~0.633; P=0.001), tumor-brain interface (OR 36.307; 95% CI 15.438~85.390; P=0.000), finger-like protrusion (OR 6.011; 95% CI 2.448~14.760; P=0.000), mushroom sign (OR 12.392; 95% CI 2.451~62.644; P=0.002), and bone invasion (OR 3.272; 95% CI 1.664~6.436; P=0.001) were independent risk factors for brain invasion in meningioma.

**Figure 3:**
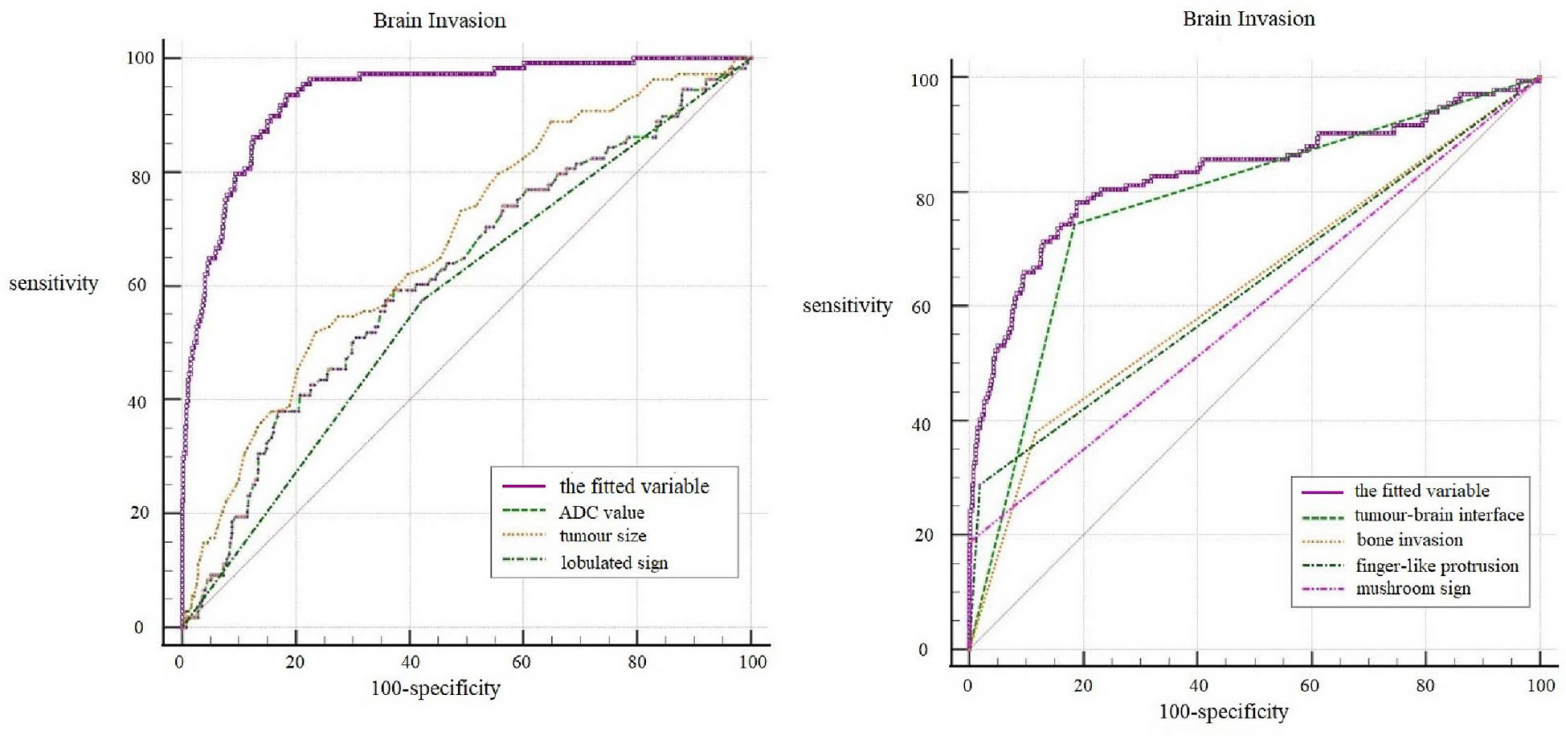
ROC curves of independent risk factors for brain invasion.

ROC curves of the seven independent risk factors for diagnosing brain invasion, including tumor size, ADC value, lobulated sign, tumor-brain interface, finger-like protrusion, mushroom sign and bone invasion, were generated (**Figure 3**). The results showed that the tumor-brain interface had the highest diagnostic accuracy for brain invasion in meningioma (AUC 0.860; 95% CI 0.832~0.885; sensitivity 0.898; specificity 0.822). Finally, the ROC curve of the fitted variable obtained by multivariate logistic regression for diagnosing brain invasion in meningioma was created (AUC 0.935; 95% CI 0.910~0.959; sensitivity 0.935; specificity 0.817), indicating good diagnostic efficiency (**Figure 3**).

## Discussion

MRI is one of the most reliable imaging methods recommended by the WHO Guidelines for the Classification of CNS Tumors (Fifth Edition 2021) for meningioma diagnosis, follow-up, and recurrence detection. MRI has high soft tissue resolution, and the sensitivity and specificity of MRI can reach 75.0% and 93.5%, respectively, for evaluating tissue conditions in and around the tumor (5). Previous literature reports have mostly focused on MRI-based predictions of meningioma WHO classification, and there have been very few independent studies regarding brain invasion in meningioma (10–15). This lack of attention to the potential assessment value of preoperative imaging for brain invasion in meningiomas tends to result in incomplete clinical surgical resection and specimen retrieval, leading to increased recurrence and mortality rates in patients after surgery, and the pathological diagnosis of brain invasion is severely underestimated as a result (9). Therefore, it is necessary to set up imaging criteria for brain invasion based on known pathological findings to distinguish whether the meningioma has invaded into the adjacent brain parenchyma. Previous brain invasion studies have included very limited MRI features and have not analyzed the diagnostic efficacy of each feature. In this study, the MRI features of meningiomas were comprehensively analyzed, and WHO grade and brain invasion were analyzed as outcomes, aiming to identify features that could predict WHO grade and brain invasion.

In our study, the male/female ratio was higher in malignant meningiomas and brain invasion than in benign meningiomas and meningiomas without brain invasion, but the incidence in males was still lower than the incidence in females, which is different from a previous report that the incidence of brain invasion in meningiomas is higher in males than in females (17). Age and T1WI and T2WI SI were not directly correlated with WHO grade and brain invasion in meningiomas, consistent with previous reports (17, 18). Adeli et al. (14)concluded that the proportion of meningiomas with brain invasion showing heterogeneous enhancement was higher than that of tumors without brain invasion, without considering their WHO grades. However, this study showed that the degree and homogeneity of enhancement cannot be used to distinguish features between benign and malignant tumors or to predict brain invasion in meningioma; we suggest that the degree and homogeneity of enhancement is related to the blood supply of the of meningioma and its composition (19). This study found that the dural tail sign and venous sinus invasion were not related to WHO grade and brain invasion, but according to Maiuri et al. (20), venous sinus invasion may be related to the location of tumor growth.

According to a previous literature report, WHO grade 3 meningiomas are larger than grade 1 and grade 2 meningiomas, with a cutoff value of 5 cm (21). Univariate regression analysis in this study also showed that the higher the WHO grade was, the larger the tumor; meningiomas with brain invasion were larger than those without brain invasion. Multivariate regression analysis showed that tumor size was also an independent risk factor for brain invasion, but the diagnostic efficacy was not high (AUC: 0.677). In this study, vascular flow void and peritumoral edema were found to be associated with WHO grade and brain invasion, and both were more common in meningiomas with brain invasion than in meningiomas without brain invasion, but multivariate regression analysis showed that neither of these features were independent risk factors for WHO grade and brain invasion. In previous studies, some scholars believed that perineural edema was predictive of brain invasion (10, 14). They suggested that perineural edema is due to the erosion of brain parenchyma by tumor tissue following destruction of the tumor-brain interface and is therefore more pronounced in those with brain invasion than in those without. It is possible that edema was an effective factor but not an independent predictor in our study because we did not calculate edema volume, and the presence or absence of edema alone may not have predicted the meningioma WHO grade and brain invasion.

Reviewing previous literature, the lobulated sign is more common in meningiomas with higher WHO grades, with the proportion of lobulated signs in anaplastic meningiomas reaching up to 100% (21). The present study showed that the lobulated sign was an influential factor for WHO grade and brain invasion in meningioma and an independent risk factor for brain invasion, a finding consistent with Adeli’s study (14). However, the efficacy of the lobulated sign in diagnosing both WHO grade and brain invasion was low (AUC: 0.557 and 0.576, respectively). Finger-like protrusion and the mushroom sign have also been reported by many scholars as characteristics of malignant meningioma, and the pathological basis of the mushroom sign is caused by tumor invasion of the adjacent dura mater, arachnoid membrane, subarachnoid space, pia mater and brain (22). We found that finger-like protrusion, the mushroom sign and bone invasion were all independent risk factors for predicting WHO grade and brain invasion in meningiomas. Although single factors had a low diagnostic efficacy, the specificity was good, so these factors could basically be considered characteristic imaging findings of brain invasion in malignant tumors.

**Figure 4:**
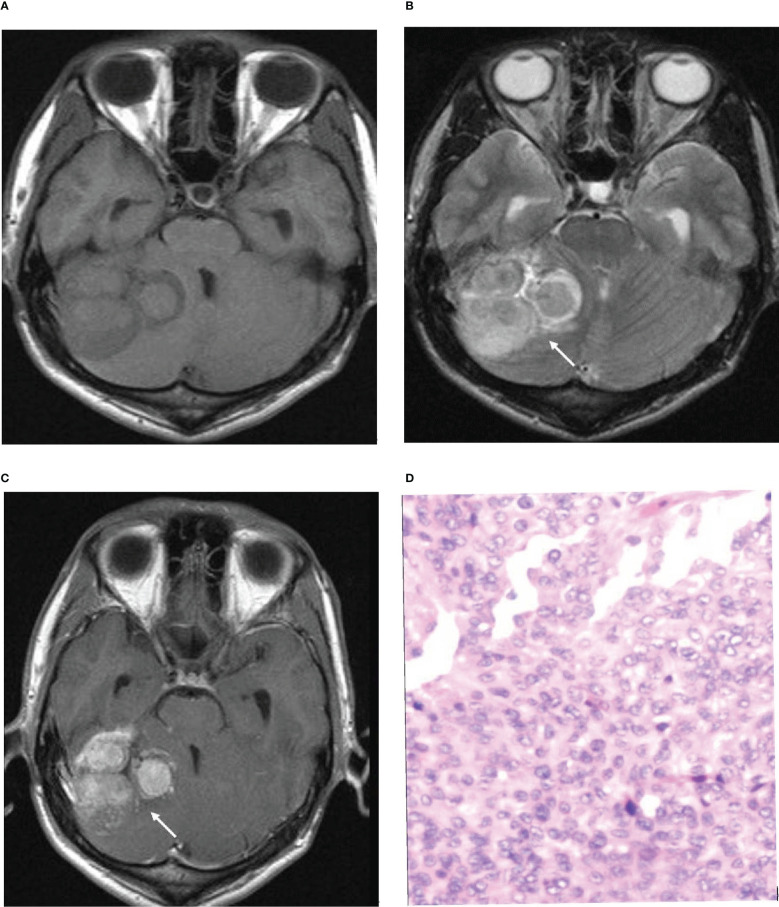
Illustrative example of the analyzed MRI variables. **(A)** Axial T1-weighted image. **(B)** Axial T2-weighted image. **(C)** Axial T1-weighted postcontrast image. **(D)** Pathological section. **(B, C)** Arrows show an unclear tumor-brain interface. **(D)**. Pathological examination confirmed brain invasion.

In the histopathological diagnosis of brain invasion in meningioma, the hematoxylin and eosin (HE) staining specimens need to contain the tumor-brain interface, and the diagnosis can only be confirmed when the tumor cells are found to be irregular and tongue-like, invading into the brain parenchyma without pia matter involvement. Therefore, it is very important to obtain specimens during the operation. In recent years, researchers have paid attention to the visualization of the tumor-brain interface in imaging studies. Adeli et al. believed that the tumor-brain interface was not correlated with brain invasion (14), while Joo et al. believed that the tumor-brain interface was important for the diagnosis of brain invasion (10, 12, 13). This study concluded that the tumor-brain interface was the most meaningful MRI feature, an independent risk factor for WHO grade and brain invasion, with the best diagnostic efficacy among all single variables (**Figure 4**). The tumor-brain interface had an AUC of 0.779 (0.746-0.810), a sensitivity of 0.742 and a specificity of 0.816 for predicting WHO grade and an AUC of 0.860 (0.832-0.885), a sensitivity of 0.898 and a specificity of 0.822 for predicting brain invasion. MRI showed excellent diagnostic efficacy overall for WHO grade and brain invasion in meningioma (AUC: 0.834/0.935). In recent years, Kandemirli et al. (11) showed an AUC of 0.74 for the diagnosis of brain invasion, and Li et al. (13) concluded that the efficacy of MRI based on clinical semantics and radiomics models had an AUC of 0.895 for predicting brain invasion. The diagnostic efficiency of MRI in the above studies is lower than that in this study, which may be because most of their studies adopted artificial intelligence computer-aided diagnosis and did not include all MRI features in the study; additionally, the number of cases was small. Moreover, some studies only included WHO grade 2 tumors, resulting in a limited reference value.

Brain invasion of meningioma is closely related to recurrence and prognosis. Perry found that the recurrence and mortality rates of benign meningiomas with brain invasion are very similar to those of atypical meningiomas, and brain invasion affects the prognosis of benign meningiomas (23). The recurrence rate of brain invasion meningioma is closely related to the degree of tumor resection. The recurrence rate of total resection is lower than that of subtotal resection and incomplete surgical resection is a direct factor of the high recurrence rate (24, 25). However, surgery requires both complete resection of the lesion and the preservation of as much normal brain tissue surrounding the tumor as possible. Therefore, preoperative imaging assessment of brain invasion is particularly important. The results of this study show that the presence or absence of brain invasion of meningiomas can be predicted preoperatively by the “tumor-brain interface”, thus allowing a fuller assessment of meningiomas with brain invasion, especially BIOB, for which complete resection is attempted during surgery, and helping to reduce their recurrence rate. In our medical center, we conducted multidisciplinary discussions on the preoperative cases suspected to be BIOB by MRI, made precise surgical plans, and removed the tumor tissue and the brain parenchyma as far as possible. After surgery, we determined whether the patients needed radiotherapy or not according to the results of intraoperative findings, pathology, immunohistochemistry and genetic testing.

In conclusion, several features of preoperative MRI are reliable in diagnosing meningioma WHO grade and predicting brain invasion, as an unclear tumor-brain interface on preoperative MRI indicates a higher WHO grade of meningiomas and a higher likelihood of brain invasion. In clinical practice, a preliminary estimate of WHO grade can be made based on the MRI features of meningiomas to predict the presence of brain invasion in advance, which helps facilitate the complete removal of lesions, guide specimen sampling, improve the accuracy of the pathological diagnosis of brain invasion, and improve patient prognosis.

## Data availability statement

The original contributions presented in the study are included in the article/supplementary material. Further inquiries can be directed to the corresponding author.

## Ethics statement

The studies involving human participants were reviewed and approved by Ethics Committee of Shenzhen Second People’s Hospital. Written informed consent to participate in this study was provided by the participants’ legal guardian/next of kin. Written informed consent was obtained from the individual(s), and minor(s)’ legal guardian/next of kin, for the publication of any potentially identifiable images or data included in this article.

## Author contributions

Conceptualization: JJ, JY, and LL. Data curation: JJ, JY, LL, KD, KZ, and FL. Formal analysis: JJ, JY, XL, and LL. Investigation: JJ, JY, XL, and LL. Methodology: JJ, JY, XL, and LL. Project administration: JJ, JY, and LL. Resources: JY and LL. Supervision: LL. Validation: all authors. Writing-original draft: JJ and JY. Writing-review & editing: LL. All authors contributed to the article and approved the submitted version.
